# Implementation of an audit with feedback knowledge translation intervention to promote medication error reporting in health care: a protocol

**DOI:** 10.1186/s13012-015-0260-y

**Published:** 2015-05-19

**Authors:** Alison M. Hutchinson, Anne E. Sales, Vanessa Brotto, Tracey K. Bucknall

**Affiliations:** School of Nursing and Midwifery, Deakin University, Melbourne, VIC Australia; Centre for Quality and Patient Safety Research, Deakin University, Melbourne, VIC Australia; Monash Health, Melbourne, VIC Australia; Center for Clinical Management Research, VA Ann Arbor Healthcare System, Ann Arbor, MI USA; School of Nursing, University of Michigan, Ann Arbor, MI USA; Alfred Health, Melbourne, VIC Australia

**Keywords:** Knowledge translation, Implementation, Audit, Feedback, Organisational context, Behaviour change, Quality improvement, Medication safety, Clinical decision-making

## Abstract

**Background:**

Health professionals strive to deliver high-quality care in an inherently complex and error-prone environment. Underreporting of medical errors challenges attempts to understand causative factors and impedes efforts to implement preventive strategies. Audit with feedback is a knowledge translation strategy that has potential to modify health professionals’ medical error reporting behaviour. However, evidence regarding which aspects of this complex, multi-dimensional intervention work best is lacking. The aims of the Safe Medication Audit Reporting Translation (SMART) study are to:

1. Implement and refine a reporting mechanism to feed audit data on medication errors back to nurses

2. Test the feedback reporting mechanism to determine its utility and effect

3. Identify characteristics of organisational context associated with error reporting in response to feedback

**Methods/design:**

A quasi-experimental design, incorporating two pairs of matched wards at an acute care hospital, is used. Randomisation occurs at the ward level; one ward from each pair is randomised to receive the intervention. A key stakeholder reference group informs the design and delivery of the feedback intervention. Nurses on the intervention wards receive the feedback intervention (feedback of analysed audit data) on a quarterly basis for 12 months. Data for the feedback intervention come from medication documentation point-prevalence audits and weekly reports on routinely collected medication error data. Weekly reports on these data are obtained for the control wards. A controlled interrupted time series analysis is used to evaluate the effect of the feedback intervention. Self-report data are also collected from nurses on all four wards at baseline and at completion of the intervention to elicit their perceptions of the work context. Additionally, following each feedback cycle, nurses on the intervention wards are invited to complete a survey to evaluate the feedback and to establish their intentions to change their reporting behaviour. To assess sustainability of the intervention, at 6 months following completion of the intervention a point-prevalence chart audit is undertaken and a report of routinely collected medication errors for the previous 6 months is obtained. This intervention will have wider application for delivery of feedback to promote behaviour change for other areas of preventable error and adverse events.

## Background

Health care is inherently complex and therefore error-prone, and despite health professionals striving for high-quality patient care, medical error and threats to patient safety are ubiquitous [1]. Although estimates of the frequency of medical errors and injuries vary considerably, even the most conservative estimates indicate that the problem is widespread [2]. Significantly, preventable medical errors are associated with immeasurable human costs and substantial economic burden [3]. Widespread underreporting of medical errors [4–10] hampers efforts to analyse causative factors and hinders ability to systematically target preventative strategies. One strategy to promote reporting, *audit with feedback*, has the potential to modify health professionals’ behaviour; however, limited evidence exists regarding which aspects of this complex, multi-dimensional intervention work best.

In this paper, we report a study protocol to refine and test an audit with feedback intervention (the Safe Medication Audit Reporting Translation (SMART) intervention). The intervention is aimed to inform decision-making and motivate behaviour change among nurses in order to maximise reporting of medication errors and ultimately improve patient safety.

### Use of audit to monitor performance and improve patient safety

In health care, auditing practice is widely used to measure performance and inform quality improvement initiatives. Collection of audit data and routine reporting of adverse events (AEs) provides evidence of performance in relation to quality standards and use of research evidence. While audit is necessary, used alone it is unlikely to be sufficient to improve quality [11]. Audit data, however, *can* be fed back to stimulate behaviour change [12, 13].

Feedback plays a key role in organisations, providing employees with information about performance that meets or fails to meet the expected standard [14]. However, feedback can have a positive or deleterious effect (such as passive resistance), dependent on how it is delivered [14]. This highlights the importance of intervention design in order to ensure that the feedback promotes and supports behaviour change to improve performance.

### Evidence for audit with feedback

Evidence indicates that the effectiveness of audit with feedback is likely to be greater when: baseline adherence to the practice is low [12, 13]; health professionals actively participate in the change process [12]; the feedback includes clear and specific goals and a strategy for achieving them [15]; it is delivered in a timely manner in association with the respective behaviour [16, 17]; the feedback is delivered both verbally and in writing, and it is delivered by a person in a position of seniority, rather than an unknown source [13]; and the feedback is delivered in repeated cycles as opposed to a single episode [13].

Authors of a 2006 Cochrane review, and the updated review in 2012, of evidence for effectiveness of audit with feedback concluded that the intervention is one for which we know little about how, when and why it is most effective [12, 13]. However, authors of the updated review concluded, “when optimally-designed and used in the right context, [audit with feedback] can play an important role in improving professional practice” ([13], p. 30). According to their findings, audit with feedback has potential to result in a small but meaningful improvement in health professionals’ behaviour [13]. Yet Foy et al. warn “… audit and feedback will continue to be an unreliable approach to quality improvement until we learn how and when it works best” ([18], p. 50).

Feedback is not a simple stimulus, but rather a complex, multi-dimensional intervention that is dependent on characteristics of the organisational context, the individual recipient, the message and the source of feedback [14, 15, 19–21]. Identifying six dimensions that impact on the form and intensity of feedback (recipient, format, source, frequency, duration and content), Jamtvedt et al. note that a lack of attention to different feedback intensities is evident in published studies [12]. Additionally, the literature provides limited description of feedback innovations in terms of the process of development and design characteristics [11, 12, 15]. Ivers and colleagues call for components of future feedback interventions to be carefully described, including the theoretical justification [15].

The influence of contextual factors on the effectiveness of feedback is also being increasingly recognised [22, 23] but remains poorly understood. Hysong and colleagues found that settings perceived as high performing were more likely to rely on chart data for feedback and placed greater emphasis on educational approaches to feedback [24]. Evidence, however, for the means by which such an intervention works in the context of complex healthcare organisations is scarce [11].

### The impact of medication errors upon quality and safety in health care

Over a decade has elapsed since the Institute of Medicine released their influential publication, *To Err is Human* [25], in which they reported that between 44,000 and 98,000 deaths occur annually as a result of medical errors. This report attracted international attention and significantly raised the profile of patient safety in hospitals and with health policy leaders [26].

While some progress has been made since publication of *To Err is Human* [25], the Australian Commission on Safety and Quality in Health Care estimates that medication administration errors occur in 5–10 % of all medicines administered, signalling an immense safety and quality issue. In England, medication administration error rates reportedly occur in 3–8 % of all hospital admissions [27], while in the USA, medication errors have been estimated to account for 7,000 deaths per year [25]. The Institute of Medicine Committee on Identifying and Preventing Medication Errors estimates that at least 1.5 million preventable adverse medication events occur annually in the USA, warning that this is likely to significantly underestimate the problem [28]. Other international research indicates that medication administration errors are associated with 5 % to 20 % of AEs [29], with 35.1 % of these being preventable [2].

### Medication errors are underreported

The international literature laments the dearth of reliable statistics globally regarding medication error rates. The aforementioned medication error rates are just the tip of the iceberg according to the Institute for Safe Medication Practices (ISMP) in the USA [30]. This is further corroborated by literature in this area, with estimates of medication error reporting rates ranging from 25 % to 70 % of all medication errors [8, 9, 31]. Researchers found that 34.8 % of nurses and physicians reported less than 20 % of perceived errors within the previous 12 months [10].

While reporting of such events is essential for promotion of a safety culture and to inform strategies to increase safety, there are numerous reasons for underreporting, including a perception that if no harm is caused, the error is not worthy of reporting [32, 33]. Lack of feedback has also been cited as one of the most common reasons for failure to report medication errors [34]. However, system modifications, feedback and education are unlikely to lead to significant improvements in reducing errors if the information surrounding errors is misleading or incomplete. It is well recognised that significant underreporting masks the true incidence of the problem and the circumstances surrounding errors, seriously hampering efforts to identify potential solutions and strategically target preventative strategies.

Medication therapy is the most common medical treatment received by patients, and medication administration is a routine part of nursing practice. Errors in medication administration expose patients to preventable harm [35]. The human costs related to medication errors encumber those who are harmed, their families and friends, as well as the health professionals who care for them. Thus there are ethical, humanitarian and financial imperatives to expose the extent and aetiology of medication errors and AEs and implement strategies to improve detection, management and prevention [3]. Evidence suggests that up to 80 % of medical errors are the result of system defects [36]. Thus, reporting of medication errors is fundamental to quality improvement efforts and redesign initiatives to promote safety in health care.

This study is designed to fill gaps identified in existing research in this field, specifically by exploring how, when and in what context feedback works best to influence medication error reporting behaviour among nurses. The findings of this study will add to knowledge about characteristics, including the content and intensity (dose) of audit and feedback that are important in influencing health professionals’ behaviour regarding reporting of medication errors. Measurement of health professionals’ perceptions of organisational context will enable us to assess the extent to which contexts perceived as more favourable are associated with increased reporting of errors.

### Aim, purpose and study objectives

This study aims to refine and test an audit with feedback knowledge translation intervention (SMART) in order to promote nurse reporting of medication-related errors in clinical practice and add to the evidence base regarding nurses’ response to audit with feedback. The clinical purpose of the intervention is to increase reporting and accuracy of medication error data so that targeted strategies may be developed to reduce their incidence in the future.

In order to achieve this, the specific aims are:

1. To implement and refine a reporting mechanism to feed audit data on medication errors back to inpatient nurses
2. To test the feedback reporting mechanism to determine its utility (including understandability, usability, usefulness and effectiveness in increasing medication error reporting)
3. To identify characteristics of organisational context associated with error reporting in response to feedback

### Theoretical framework

This study is informed by two complementary theoretical approaches. The Promoting Action on Research Implementation in Health Services (PARIHS) framework focuses on the relationship between three key elements (evidence, facilitation and context) in influencing the implementation of research in practice [37, 38]. This framework, however, has limited applicability in studying the influence of individual characteristics on behaviour change. For this reason, the Theory of Planned Behaviour (TPB), which helps explain an individual’s intent to change their behaviour, also informs this study.

#### PARIHS

The PARIHS framework was developed by Kitson et al. to help explain the reasons for success or failure of implementation projects [39]. For the purposes of this study, *evidence* relates to data on medication error and reporting rates, *context* constitutes the ward environment (i.e*.*, culture, leadership and evaluation/feedback processes) that impact upon medication error reporting and *facilitation* denotes roles and strategies to promote the behaviour change.

#### TPB

The TPB is an extension of the Theory of Reasoned Action [40], according to which behavioural intention is the precursor to actual behaviour and intentions are formed based on information or beliefs that a certain behaviour will lead to a particular outcome [41]. Subjective norms, the perceived social pressure to comply with a behaviour, can determine whether or not an individual performs the behaviour [42].

The TPB extends on the Theory of Reasoned Action to include individuals’ perceptions of behavioural control [43]. Perceived behavioural control, perception of the ease or difficulty of performing the behaviour, is thought to influence both behaviour and intent [43]. Inclusion of perceived behavioural control expands understanding of the constraints on behaviour and acknowledges that intentions do not always predict behaviour [43]. This is relevant to medication error reporting behaviour in that intent to report errors may be influenced by other factors that ultimately lead to non-reporting behaviour. It is also important to determine if feedback affects behavioural intent. Ajzen [42] suggests that these constructs (beliefs, subjective norms and perceived behavioural control) will vary in their level of influence across settings and behaviours; for example, in settings where individuals have strong opinions and attitudes perceived behavioural control may be a less reliable predictor of intention [43]. A meta-analysis of 161 studies using the TBP [43] supported the use of this theory to predict intentions and behaviour.

### Pilot testing an intervention to improve medication error reporting

Consistent with the UK Medical Research Council guidelines for the development of complex interventions [44], we conducted a small study in a 32-bed acute care ward at a large private hospital to inform development and undertake pilot and feasibility testing of medication chart audit tools and the SMART feedback intervention.

Baseline audit data were collected on key aspects of medication administration. At baseline, all ward nursing staff were invited to complete the Alberta Context Tool [45, 46], a questionnaire designed to elicit perceptions of the work context (including culture, leadership and feedback mechanism). The feedback intervention comprised Microsoft PowerPoint^©^ presentations to display audit data and survey results in graph form, coupled with face-to-face discussions about the results. Additionally, copies of the results slides were displayed on the ward for staff to view. The feedback was augmented with information about the definition of a medication error, the importance of reporting medication errors and near misses and the importance of a no-blame culture.

Medication administration audit data were collected 2 months following the feedback intervention, and staff were re-surveyed about their perceptions of the work context 6 months following collection of the baseline data. The frequency of medication errors in the 12 months prior to the intervention and in the 6 months post-intervention was compared. We found an 80 % increase in the reporting of medication errors and a statistically significant improvement in perceptions of evaluation and feedback mechanisms (including access to data, review, monitoring and benchmarking). The considerable increase in frequency of error reporting in the post-intervention phase highlighted the potential for this intervention to improve error reporting across the organisation and beyond and the importance of more rigorous testing of the intervention.

This pilot work underpins the study that is the subject of this protocol. The premise upon which this study is built is that comprehensive reporting of medication errors is necessary to inform interventions and process redesign to prevent further errors occurring.

## Methods

This controlled interrupted time series study design incorporates two pairs of matched wards at an acute care hospital with randomisation of two wards to the control group and two wards to the intervention group. Nurses in the intervention group will receive the SMART intervention (feedback of medication error audit data).

We will adopt the National Coordinating Council for Medication Error Reporting and Prevention standard definition of a medication error: “any preventable event that may cause or lead to inappropriate medication use or patient harm while the medication is in the control of the health care professional, patient, or consumer” [47].

### Setting and sample

This study will take place at a large, not-for-profit Australian private health provider. Four acute care wards will be included in the study. The wards will be matched as closely as possible according to clinical casemix (diagnostic group and average length of stay).

A key stakeholder reference group will be established to determine the preferred feedback methods and mechanism (the design of the feedback report and how it is presented to staff). This group was instrumental to feedback design in the pilot study and will include clinical nurses and representatives from nursing management, pharmacy and medicine (*n* = 10).

Managers (*n* = 4) and nurses (*n* = 160) working in the four participating wards will be invited to participate in this study.

### Data sources

#### Audit data sources

Two sources of audit data will be used in the feedback report:

1. Medication error reports and medication adverse event data that are routinely reported in the risk management and reporting system (RiskMan.Net ©) in the study organisation and
2. Data generated from point-prevalence audits of medication documentation in patients’ medical records.

#### Survey data sources

Two other sources of data are surveys of nurse participants:

1. The Alberta Context Tool (ACT) will be administered twice to nurses in all four wards: at baseline, prior to introduction of the feedback reports and 12 months following baseline data collection [45, 46].
2. The post-feedback survey will be administered four times to intervention ward participants (1 week following each feedback report) [11].

#### Interview data sources

Following the final 2 cycles of feedback, focus group interviews will be conducted with consenting nurses working on the intervention wards.

### Instruments

The ACT was developed, and tested for validity, for use in acute care settings [45]. The ACT consists of a suite of instruments designed to assess *modifiable* characteristics of organisational context [48]. The tool consists of 56 items to assess eight dimensions of the work context: culture, leadership, evaluation, social capital, informal interactions, formal interactions, structural and electronic resources, and organisational slack (representing time, space and human resources).

The *post-feedback survey* was developed to elicit perceptions about the use of feedback, including whether the individual received the feedback report, whether they reviewed it, whether and how they used it to inform their practice, and barriers to using the report [11]. Additionally, based on the Theory of Planned Behaviour, a series of questions assess the individual’s intent to change behaviour in response to the feedback, and if so, how [11]. The instrument also includes questions to assess the utility of the report, including whether the feedback is understandable and engaging, relevant to policy and practice, presented in a form that can be used to inform and influence practice, and whether the process of report delivery is appropriately timed and the report is readily accessible. Respondents are also invited to record suggestions/recommendations to improve the feedback reports.

The *clinical chart audit tool* developed for and used in our pilot testing will be adapted for collection of the point-prevalence data in the current study. Chart audit data will be collected on all four wards, quarterly for the 12 months.

*Focus group interviews* will be undertaken using an interview guide to elicit nurses’ perceptions and experiences in receiving, reading, discussing, assimilating and using the feedback reports to inform their actions.

### Procedure

#### Baseline data collection

The Alberta Context Tool will be administered to all nurses on all four wards prior to implementation of the intervention. Point-prevalence audit data will be collected at baseline in all four wards, and a report of medication errors recorded for each ward for the previous 12 months will be extracted from RiskMan.

#### Development of the SMART intervention

Audit data will be analysed and reported descriptively (including frequencies by time of day, day of week, type of medication, route of administration). Development of the feedback report (including the presentation and content) will be informed by recommendations and feedback from the key stakeholder reference group. Because we will have a range of variables that could be reported on, we will elicit from the key stakeholder group the priorities for reporting of data.

The format adopted for presentation of medication error report rates and medication AE rates will take into consideration: content (which variables to report), frequency, presentation (graphs, description) and supporting processes (information sessions). In developing the feedback reports, we will seek advice from the key stakeholder group regarding presentation of data, including promotion of visual appeal (considering layout, use of white space, colour, use of illustrations/figures), striving to make the content understandable (clear and concise description and illustrations that can be readily understood), and ensuring that the content is relevant.

#### Testing the SMART intervention

##### Administration

In conjunction with the key stakeholder reference group, the timing and mechanism for delivery of the feedback report will be determined. At a mutually convenient time, the researcher will meet with the relevant nurse unit managers of the two intervention wards to explain the project and arrange a suitable time for delivery of the feedback reports. A brief information session will be conducted in conjunction with delivery of the first feedback report, at a time and location determined by the nurse unit manager. Feedback reports will be provided on a quarterly basis for 12 months. The “usual care” wards will not receive the SMART intervention.

##### Evaluation

For 12 months, routinely reported RiskMan data will be extracted weekly and point-prevalence chart audits will be conducted quarterly on all four wards. On the intervention wards, data will be collected to evaluate the feedback reporting process and to elicit the opinions of managers and nurses with respect to the understandability, usefulness and usability of the report content. The findings will inform modifications to the report for future waves of report delivery.

On a quarterly basis, 1 week following delivery of the feedback report, nurses will be invited to complete the post-feedback survey, a brief, paper-based survey [11]. The researchers, in conjunction with the key stakeholder reference group, will consider the findings of the survey and modify future feedback reports accordingly. These modifications will then be re-evaluated by survey. Thus, a series of feedback reports, each containing the most current medication error report rates (from the point-prevalence medication chart audits) and medication-related errors and AEs (from RiskMan data) will be produced and each will be informed by survey data evaluating the previous reports. If and when the survey data no longer indicate the need for future modifications, the feedback report will not be modified and the staff will not be re-surveyed. Completion of the survey will be voluntary, and return of the survey will imply consent. Survey data will be manually entered into an IBM SPSS Statistics [49] database for analysis. Following the final 2 cycles of feedback, nurses on the intervention wards will be invited to participate in a focus group interview to discuss the process of using the feedback.

##### Process measures

Process measures include self-reported uptake of feedback reports (survey data), intent to change behaviour (survey data) and perceptions of the feedback report (survey data on report content, presentation; interview data).

##### Outcome measures

Outcome measures include point-prevalence audit data and medication error reports. While we expect to observe an increase in medication error reporting rate as a result of the intervention, at some point, potentially as a culture of medication safety is developed, we may observe a decrease in medication error reporting because the true rate of errors decreases.

### Post-intervention data collection

**Fig. 1:**
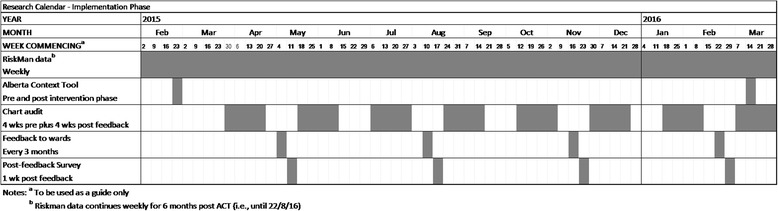
Elements and stages of data collection

Following the 12-month intervention period, the ACT will be administered to all nurses on all four wards. Six months following completion of the intervention phase (18 months post-baseline data collection), a repeat point-prevalence chart audit will be undertaken and a report of reported medication errors and AEs for the previous 6 months will be extracted from RiskMan. These data will be used to assess sustainability of the intervention. Fig. 1 illustrates the stages of the data collection.

**Fig. 2:**
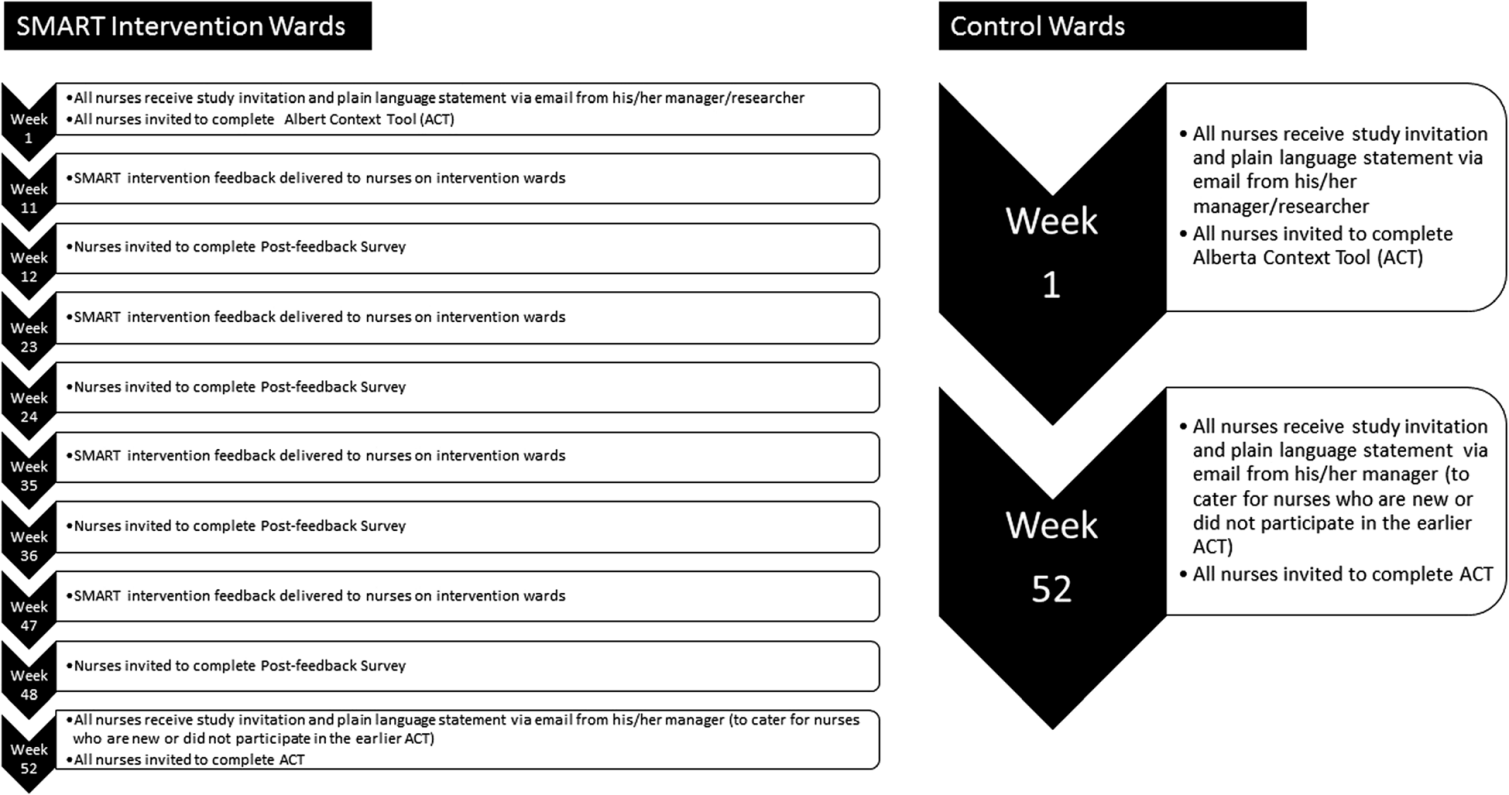
Nurse involvement in the study

Individual nurse participant involvement in the study for intervention and control wards is illustrated in Fig. 2.

### Analysis

Quantitative methods will be used to analyse the survey data. Analysis will involve the use of simple descriptive statistics, including frequencies and cross tabulations, and bivariate analysis using correlations. Interrupted time series analysis will be used to assess for change over time in response to the feedback. Outcomes will include error rates identified during the point-prevalence chart audits and the error report rates identified from RiskMan data. These data will be aggregated at ward level.

We will also examine RiskMan data for the 12-month period prior to the intervention. Intervention dose will be the main predictor, operationalised as the number of nurses on the ward who self-report reading the feedback reports. Aggregated at ward level, scores for the eight dimensions of organisational context will also be included as predictors of medication error reporting rates. Multivariate regression analyses will include cluster correction to adjust for ward and site effect. Content analysis will be used to analyse string variable responses to open-ended questions about suggestions to improve reports. Qualitative data will be analysed using content and thematic analysis techniques.

### Ethical considerations

Operational approval for the study has been provided by the Director of Nursing at the hospital. Ethics approval has been obtained from the hospital’s Human Research Ethics Committee and Deakin University. Prior to commencement of the study, permission will be sought from the Nurse Unit Managers of the selected wards in order to access staff. This is a minimal risk study.

### Expected outcomes

We expect that this knowledge translation feedback intervention will result in improved quality and frequency of reporting of medication errors. This will enable us to understand the scale and nature of medication errors occurring in the intervention units. Our intent is to produce a parsimonious, efficient and effective method of providing feedback to health professionals about a quality-related measure of practice. We anticipate that this project will result in the development of a feedback approach that can be adapted for and evaluated in other healthcare settings and for additional areas of preventable adverse events. Importantly, this study is designed to add to the body of knowledge about how, when and in what context feedback works best.

### Strengths and limitations of study

This study will add to the evidence base on use of audit with feedback among nurses. In particular, it will promote understanding of the potential for feedback to promote behaviour change regarding medication error reporting, how and in what forms nurses prefer to receive feedback and how the contexts in which nurses work affect their ability to report medication errors. Additionally, the study will contribute knowledge about the accuracy of medication error reporting, comparing hospital incident reporting system data with evidence of administration errors in medical record documentation.

Limitations of this study need to be acknowledged. The study is to be conducted at a single site, with a relatively small sample. While we will match intervention and control wards as closely as possible on the basis of clinical casemix, there is no guarantee that matched wards within the same hospital will be entirely similar (e.g*.*, differences in ward culture, patient acuity/type, staffing turnover); hence, we will adjust for differences using the available data. Feedback will be constructed based upon advice from the key stakeholder group. It is possible that this format will not suit all nurses, and so, some staff may not find the feedback meets their needs and may discount it as a result. Behaviour change in reporting may also occur as a result of other variables (such as policy changes and/or serious medication incidents that make staff more aware/anxious about reporting). Hence, not all behaviour change may be attributable to the feedback intervention. Potential for contamination of the control group exists; however, the organisation has a stable nursing workforce with a vacancy rate of less than 5 %. Thus, permanent staff do not commonly work across different wards, thereby limiting the risk of contamination.

### Dissemination and spread

This study will add new knowledge that will be of particular interest and relevance to health professionals and administrators. We will use a multi-faceted approach to disseminate the findings of this study to health professionals, administrators and policy-makers. We will present the results in journal articles and at local, national and international healthcare conferences. We will also communicate the findings through our formal and informal networks, nationally and internationally.

The partner organisation and members of the research team are committed to sharing new knowledge to promote safe, high-quality care delivery. Our findings will be disseminated through the collaboration’s existing networks, and a summary of the findings and information on the study, in a plain language format, will also be posted on the university website. Members of the research team sit on a range of policy decision-making committees and professional associations. As such, they have extensive networks for wide dissemination of the study findings.

### Study status

Data collection commenced in February 2015 and will finish in 2016. The data collection cycle is illustrated in Fig. 1.

## Discussion

This study has the capacity to make a significant contribution to the literature in terms of the efficacy of audit with feedback for nurses. Factors affecting reporting of medication errors will be clarified and will allow healthcare organisations to target quality programmes and feedback strategies to effect positive change. The theoretical basis and methodological design of this study integrates well-established theories and methods to comprehensively address the research questions. This intervention will potentially have wider application for the delivery of feedback to health professionals working in other settings, for other areas of preventable adverse events and to promote behaviour change in aspects of care such as use of clinical practice guidelines. These findings will inform development of future interventions, including system redesign, to prevent medication errors. This research has potential to inform initiatives to improve the quality and safety of care received by patients in the acute care setting and will also have significance internationally in the field of knowledge translation.

## Abbreviations

ACT: Alberta Context Tool
AE: Adverse event
PARIHS: Promoting Action on Research Implementation in Health Services
SMART: Safe Medication Audit Reporting Translation
TPB: Theory of Planned Behaviour
